# The effect of distraction techniques on pain and stress during labor: a randomized controlled clinical trial

**DOI:** 10.1186/s12884-019-2683-y

**Published:** 2019-12-30

**Authors:** Paria Amiri, Mojgan Mirghafourvand, Khalil Esmaeilpour, Mahin Kamalifard, Reyhaneh Ivanbagha

**Affiliations:** 1 Department of Midwifery, School of Nursing and Midwifery, Tabriz University of Medical Sciences, Tabriz, Iran; 2 Social Determinants of Health Research Centre, Faculty of Nursing and Midwifery, Tabriz University of Medical Sciences, Tabriz, Iran; 3 Nursing and Midwifery Faculty, Midwifery Department, Tabriz University of Medical sciences, Tabriz, Iran; 4 Faculty of Education and Psychology, Tabriz University, Tabriz, Iran; 5 Department of Midwifery, School of Nursing and Midwifery, Tabriz University of Medical Sciences, Tabriz, Iran; 6 Department of Midwifery, Faculty of Nursing and Midwifery, Khlkhal University of Medical Sciences, Khalkhal, Iran

**Keywords:** Distraction techniques, Pain, Fear of childbirth, Stress, Delivery

## Abstract

**Background:**

Pain control and the stress associated with labor and delivery are among the most important issues of health care system. Use of distraction techniques during childbirth is reported to reduce pain and stress of labor. However, there is a limited publication that looked on the effect of distraction techniques on labor pain and stress. Thus, the aim of this study was to determine the effect of distraction techniques on labor pain and stress (primary outcomes), fear of childbirth, length of delivery stages, first minute Apgar score and oxytocin consumption (secondary outcomes).

**Methods:**

A randomized controlled clinical trial was conducted on 68 pregnant women. Participants were allocated into two groups (intervention and control groups) by blocked randomization. Participants in the intervention group received distraction techniques in four sessions. Questionnaires of Fear of Childbirth (W-DEQ version A) and PSS once were completed before intervention and again at the 36th week for the W-DEQ and in the active phase for the PSS through interviews. The pain was assessed through VAS during active phase per hour. The length of delivery stages was recorded in the partograph chart. Data were analyzed by independent t-test and ANCOVA.

**Results:**

The mean of perceived stress during labor in the intervention group was significantly less than that of the control group (AMD: -3.2; 95% CI: − 0.8 to − 6.0; *P* = 0.01). The mean (SD) of pain intensity during labor was less than in the intervention and control groups compare to the control group (6.2 vs 7.5; *P* < 0.001). There was no significant difference between the two groups in terms of fear of childbirth score (AMD: 5.3; 95% CI: 13.0 to − 6.0; *P* = − 2.3). Moreover, there was no statistically significant difference between the two groups in terms of the active phase of labor (*P* = 0.504), second stage of delivery (*P* = 0.928), total length of delivery (*P* = 0.520), Apgar score (*P* = 1.000) and frequency of oxytocin consumption (*P* = 0.622).

**Conclusion:**

According to the results, distraction techniques can reduce the pain and stress of labor, but further studies by using the distraction techniques are needed to reach a decisive conclusion.

**Trial registration:**

IRCT2017042910324N39; Name of registry: Iranian Registry of Clinical Trials; Registered 11 September 2017. URL of registry: https://fa.irct.ir/user/trial/10814/view. Date of enrolment of the first participant to the trial: September 2017.

## Background

Physiological reactions to pain notify a person of a dangerous biological agent in the body [1]. However, labor pain is not a pathologic factor and rather is a physiological condition due to contraction of the smooth muscles of the uterus to guide the fetus and other contraceptive products out of the body. In this regard, the intensity of this pain is unique [2] such that it is reported as the worst pain in the world. In some cases, the severity of labor pain is so much that it is compared even with the breakdown of the fingers [3]. Therefore, its control has been one of the most important healthcare goals worldwide [4]. Various factors can affect the severity of pain such as experience, fear, anxiety, race, cultural, social, and environmental factors, demographic, and biological characteristics [5] .In addition to pain, childbirth is a critical psychological, social, emotional, and tangible event and a prerequisite for every woman [6].

Childbirth stress is referred to any stress or anxiety of the mother about the course of delivery and pregnancy [7]. Mother’s contact with stressful agents during pregnancy can cause adverse outcomes such as low-weight birth, preterm labor, and spontaneous abortion. Also, stressors increase the catecholamines and cortisol levels and can suppress the immune system [8]. Mental stress, anxiety, fear of labor pain, the unknown space of the labor room and lack of trust in its staff can contribute to increased labor length and the proliferation of pain through secretion of catecholamines, cortisol, and epinephrine to overcome these tensions [9].

Fear of childbirth is defined as negative perceptions in mothers that influence by various reasons such as mother’s personal characteristics [10]. Fear of childbirth can also be associated with other psychological tensions such as feeling pain more severe than the actual level [11], prolonged labor length [12], and depression [13]. Severe childbirth fear increase elective cesarean section and may follow by an increase in the complications of cesarean delivery on mother and baby [14], that cause a financial burden on family and state and increase hospitalization time and, as a result, filling beds in the hospital [15]. To date, various pharmacological and non-pharmacological approaches have been proposed for controlling and reducing pain [16]. Distraction is one of these techniques that has attracted more attention from researchers and medical communities over the past 5 years, specifically in the field of dentistry and phlebotomy in children [17].

The distraction technique is a cognitive-behavioral approach [18] that is used to control emotions. This technique can distract a person’s mind of stress, fear, anger, and discomfort. The logic of using this method is that our mind has the feature that it cannot think twice at the same time. When we leave our minds at the height of our excitement, the mind is distracted from the excitement by the senses [19]. Also, the distraction technique decreases the effect on the central nervous system and pain-free nerve transmitters [20]. Some techniques of this method include counting numbers, remembering poetry, remembering a pleasant memory, recalling a joke, not thinking [19], using vulgar cards [21], using virtual reality [22], and watching TV [23]. Distraction technique has been used to measure the amount of pain in children [24], iodization [25]. burn [26], colonoscopy [27], and to control anxiety in patients referred for episiotomy, IUD insertion, hysteroscopy, and uterine aspiration. Moreover, this technique has been used in dealing with an endometrial biopsy [22], fears of having a portal vein in cancer patients [28], and fear of receiving hyperbaric oxygen [29].

Considering the effects of pain, stress, and the fear of childbirth on mother and baby and limited publication on the effect of distraction techniques on the pain and stress of labor, the present study was conducted to determine the effect of distraction techniques on labor pain and stress (primary outcomes) and fear of childbirth and the length of delivery stages (secondary outcomes).

## Methods

CONSORT guidelines were adhered for reporting of this trial.

### Study design

This study was a single-blind randomized controlled clinical trial with two parallel group (intervention and control groups). This study approved by the Ethics Committee of Tabriz University of Medical Sciences (code: IR.TBZMED.REC.1396.453). The population of this study includes pregnant women with gestational age of 28–32 weeks who referred to health centers of Khalkhal city, west Azerbaijan province, Iran from February to September 2018. All participants signed a written informed consent form. Only data analyser was blinded to the intervention received by the study groups.

### Study population

The inclusion criteria were being pregnant with gestational age 28–32 weeks, having the first or second pregnancy, the willingness to delivery in Khalkhal’s Imam Khomeini Hospital, and lack of participation in the same classes. The exclusion criteria were high-risk pregnancies including gestational diabetes, preeclampsia, twin and multiple pregnancies, mothers with amniotic fluid and placenta disorders, fetal death, mental illnesses, and taking certain medications, having cesarean section indications, previous cesarean section, abnormal fetal presentation, pelvic stenosis, and fetal macrosomia.

### Randomization & masking

Participants were assigned to two groups of intervention (recipient of distraction techniques) and control through stratified block randomization based on the number of deliveries (first delivery and second delivery) with block sizes of 4 and 6 and with a 1:1 assignment ratio. Blocking was done by a non-involved person in data collection and analysis. To conceal the allocation, the type of intervention was written on a sheet of paper and sealed in matte envelopes. Envelopes were opened by the researcher in the order of entry of the participants to the research and the type of group was identified.

### Procedures

Sampling started after receiving the ethics code from the Ethics Committee of Tabriz University of Medical Sciences and registering the study on the Iranian Registry of Clinical Trials site. Khalkhal, a city in northwestern Iran, has three health centers. During the sampling process, the researcher inquired about the information of pregnant mothers during the week of 28–32 through the Integrated Health System and contacted the mothers who had some inclusion criteria. Also, over a telephone call, the researcher briefed the research plan and its objectives. The participants were assessed based on the eligibility criteria and, if they were eligible and willing to participate in the study, asked to attend a health center at a specific time. In attendance, comprehensive data including the goals, importance, and benefits of participation in the study, as well as the stages of the implementation of the research were provided to pregnant women. Moreover, if they desired, the basic questionnaires including socio-demographic characteristics questionnaire, PSS and fear of childbirth questionnaire (W-DEQ version A) were completed through interviews and participants were allocated into two groups.

Counseling based on distraction techniques for controlling stress, fear, and the pain was presented to participants in the intervention group in four sessions within a week. All counseling sessions were conducted by the first author. The first session was held on the 32nd week of pregnancy. During this meeting, all participants were trying to establish friendly relations and gaining the confidence of the participants. Then, the researcher described in detail the definition of distraction technique and how it affects pain management. In the second session, several distraction techniques were explained for the intervention group; i.e., watching movies, solving table and puzzles, listening to music, illustrating child’s future, remembering memory, talking about their skills, reverse counting the numbers, counting the serum drops used during labor, and also about personal interests and experiences. In the third session, the researcher received feedback from the previous session, exercises were performed at home, and the participants were asked to do exercises including counting certain letters while watching the video and playing music, reverse counting the numbers 3 by 3 out of 1000, record the length of time they can entertain themselves with these methods, and record their interests. Based on their records, the researcher provided necessary facilities for them to use during labor. In the fourth session, stages of delivery, delivery progress, control of stress and fear using distraction techniques, birth space, and childbirth preparation were discussed, and feedback from the intervention group was received. All participants in the intervention and control groups were asked to attend the health center at week 36 and the W-DEQ was completed by interviewing them. For participants in the control group, after the completion of the W-DEQ, training was given about signs of delivery, the stages of delivery and the appropriate time for a referral to the hospital. In all sessions, the principles of counseling were thoroughly followed. Then, all the participants were given a phone number of the researcher to contact the researcher in the event of labor pain and referral to the hospital. The researcher attended the hospital and used the VAS scale to record the pain of the participants every hour during the active phase of labor. Then, the perceived stress questionnaire was completed after the participant’s admission in the delivery department at the beginning of the active phase of labor through interview. The intervention group, based on their interest in the third session, was provided by distraction facilities such as movies, music, table, puzzle, book, and more. The researcher was active alongside the mother during the entire phase of the active phase and all distraction techniques by the participants including reverse counting the numbers, counting the serum drops, memorizing, illustrating, etc. were conducted in the presence of the researcher. For mothers, their favorite film was played and they were asked to carefully watch the movie and count certain letters when watching them. The control group received routine care.

### Primary outcomes

The primary outcomes included severity of labor pain and perceived stress that was measured by VAS and PSS, respectively before intervention and in the active phase of labor. VAS is a graded ruler of 10 cm in length, in which the patient should determine his own assessment of pain on this graded line from zero (painless) to 10 (the most extreme pain imaginable). Based on this scale, the score zero denotes the labor without pain, 1–3 as mild, 6–4 as average, 9–7 as severe, and 9–10 as very severe pain levels [30]. PSS consists of 14 items and scores are based on 5-item Likert as follows: never = 0, almost never = 1, sometimes = 2, often = 3, and many times = 4 points. The items 4–5–6-7, 9, 10, and 13 are scored inverse (never = 4, many times = 0). The lowest score is 0 and the highest score is 56. A higher score indicates more perceived stress [31] . The reliability of the Persian version of this questionnaire was calculated by Bastani et al., by the internal consistency method. They obtained a Cronbach’s alpha coefficient of 74% for this questionnaire [32].

### Secondary outcomes

The secondary outcomes included fear of childbirth, duration of active phase of labor and second stage of delivery, total length of delivery, first minute Apgar score and oxytocin consumption. The W-DEQ-Version A was used to assess the fear from childbirth before intervention and again at the 36th week of pregnancy. This questionnaire has 33 questions. Mothers identify their personal feelings based on a 6-item Likert scale (at all = 0, very low = 1, low = 2, average = 3, high = 4, and very high = 5). Questions 1, 4, 5, 9, 10, 13, 14–16–17-18, 21–22-23, 26, 28–29-30 are scored in reverse. The score range is 0 to 165 and a higher score indicates more fear [33]. The reliability of the Persian version of this questionnaire was assessed by Abedi et al., who reported the Cronbach’s alpha of 0.64 [34].

The length of delivery stages, first minute Apgar score and oxytocin consumption were recorded in the partograph chart during labor and childbirth by researcher.

### Statistical analysis

The sample size in this study was calculated based on both pain and stress variables using G-Power software. According to the results of the study by Madadi et al. (2016) regarding the pain variable, taking m_1_ = 8.9 (pain before the intervention), m_2_ = 7.9 (pain after intervention), sd_1_ = 1.2, sd_2_ = 0.9, α = 0.05, and Power = 95% were calculated to be 31 [35]. Based on the results of Mirghafourvand et al. (2014) on the perceived stress variable and taken into account m_1_ = 26.2 (perceived stress before intervention), with a 20% reduction in mean perceived stress score due to the intervention (m_2_ = 19.65), sd_1_ = Sd_2_ = 5.5, α = 0.05 and power = 95% was calculated to be 28 [36]. Since the sample size was calculated based on the pain variable was more, considering the 10% attrition, the final sample size was calculated to be 34.

Statistical analysis of the present study was conducted using SPSS 24 software. The normality of quantitative data was investigated using the Kormogrov-Smirnov test. The results showed that the duration of the second stage of delivery and the pain score did not have a normal distribution*. The Chi-square, Chi-square for trend, Independent t and Fisher’s exact tests* were used for assessing the consistency of the two groups in terms of socio-demographic characteristics. To compare the mean perceived stress score and childbirth fear, independent t-test was used before intervention and ANCOVA test with adjustment of baseline values, and stratification factor (first delivery or second delivery) after the intervention. To compare the duration of the active phase and the total length of delivery, independent t-test was used and to compare the length of the second stage of delivery and the mean pain during labor in the two groups, Mann-Whitney U test was used. Chi-square test was applied for comparing frequency of oxytocin consumption in the two intervention and control groups. Fisher’s exact test was used to compare the Apgar score at the first minute (Those who had cesarean section, their first-minute Apgar score was assessed in the operating room). The significance level for statistical tests was considered less than 0.05. All analyses were performed based on intention to treat.

## Results

**Fig. 1:**
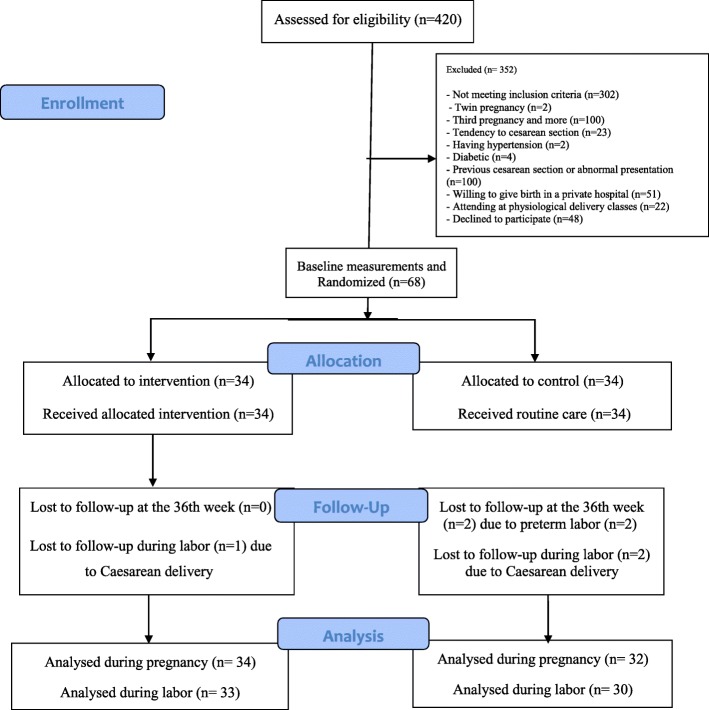
Flowchart of the study

Of the 420 pregnant mothers referred to health care centers for antenatal checkup, 118 were eligible for inclusion in the study, 68 of whom agreed to participate in this study (Fig. 1).

Table 1 shows the socio-demographic information of participants in two study groups. There was no significant difference between the two groups in terms of socio-demographic information (Table 1).

**Table 1:** Comparison of socio-demographic characteristics of the participants in two groups of receiving distraction techniques and control

Variable	Distraction Techniques *n* = 34	Control *n* = 34	*P*
Age (years)*	26.2 (5.4)	27.0 (5.6)	0.568^d^
Body mass index (kg/m ^2^)*	22.9 (2.9)	22.8 (3.6)	0.965^d^
Education			0.628^a^
Illiterate	1 (2.9)	0 (0)	
Primary school	0 (0)	2 (5.9)	
Secondary school	4 (11.8)	6 (17/6)	
High school	2 (5.9)	2 (5.9)	
Diploma	9 (26.5)	5 (14.7)	
University	18 (52.9)	19 (55.9)	
Job			0.528^c^
House wife	29 (85.3)	27 (79.4)	
Employed	5 (14.7)	7 (20.6)	
Spouse education level			0.643^a^
Illiterate	1 (2.9)	0 (0)	
Primary school	0 (0)	2 (5.9)	
Secondary school	4 (11.8)	6 (17.6)	
High school	2 (5.9)	2 (5.9)	
Diploma	9 (26.5)	5 (14.7)	
University	18 (52.9)	19 (55.9)	
Spouse job			0.863^c^
Unemployed	0 (0)	0 (0)	
Employee	8 (23.5)	8 (23.5)	
Manual worker	6 (17.6)	7 (20.6)	
Shopkeeper	7 (20.6)	5 (14.7)	
Others	15 (44.1)	12 (35.3)	
Sufficiency of monthly income for living expenses			0.165^a^
Enough	20 (58.8)	18 (52.9)	
Fairly sufficient	13 (38.2)	9 (26.5)	
Inadequate	1 (2.9)	7 (20.6)	
Wanted pregnancy			0.340^b^
Yes	31 (91.2)	29 (85.3)	
No	3 (8.8)	5 (14.7)	
Marital satisfaction level			0.455^a^
Totally satisfied	31 (91.2)	29 (85.3)	
Relatively satisfied	3 (8.8)	5 (14.7)	
Totally dissatisfied	0 (0)	0 (0)	
Gravida			0.432^c^
1	20 (58.8)	18 (52.9)	
2	14 (41.2)	16 (47.1)	

^a^Chi-square for trend test

^b^Fisher's exact test

^c^Chi-squared test

^d^Independent t-test

Variables were reported as numbers (%), except for cases * reported as mean (Standard Deviation)

### Primary outcomes

Before intervention, the mean (SD) of the perceived stress score was 15.1 (8.0) in the distraction techniques group and 15.6 (1.6) in the control group. Moreover, at the beginning of the active phase of labor, the perceived stress score was 11.8 (5.7) and 15.2 (7.1) in the intervention and control groups, respectively. Before the intervention, there was no statistically significant difference between the two groups (*P* = 0.717), but in the active phase of labor based on ANCOVA test and with adjusting the baseline score, the mean score of perceived stress in the distraction techniques group was significantly less than the control group (AMD = − 2.3; 95% CI: − 0.6 to − 0.8, *P* = 0.01) (Table 2).

**Table 2:** Comparison of perceived stress and fear of childbirth between two groups of the distraction technique and control

Variable	Distraction Techniques Mean (SD^a^)	Control Mean (SD^a^)	Mean Difference (95% Confidence Interval)	*P*-value
Perceived stress (Score range: 0 to 56)				
Before intervention	15.0 (8.0)	15.6 (6.1)	0.6 (−2.9 to 4.1)	0.717
After intervention	11.8 (5.7)	15.2 (7.1)	-3.2 (−6.0 to −0.8)	0.01
Fear of Childbirth (Score range: 0 to 165)				
Before intervention	38.3 (22.9)	46.3 (17.1)	7.9 (17.9 to −2.0)	0.117
After intervention	29.1 (18.9)	39.1 (29.6)	5.3 (13.0 to −2.3)	0.170

Independent t-test was used to compare the groups before intervention and ANCOVA test with baseline control was used after intervention. The higher the score of stress and fear, the more stress and fear

^a^Standard Deviation

The mean (SD) of labor pain severity was 6.2 (1.4) in the distraction techniques group and 7.5 (1.4) in the control group, which was significantly lower in the intervention group than in the control group (*P* < 0.001) (Table 3).

**Table 3:** Comparison of the duration of delivery stages and pain score between two groups of the distraction techniques and control

Variable	Distraction techniques		Control		*P*-value
	Med (Per 25 to Per 75)^a^	Mean (SD^b^)	Med (Per 25 to Per 75)^a^	Mean (SD^b^)	
Active phase (Minute)	240.0 (307.5 to 180.0)	246.6 (131.3)	260.0 (180.0 to 260.0)	268.7 (130.9)	0.504^c^
Second stage (Minute)	25.0 (15.0 to 40.0)	27.8 (15.1)	30.0 (13.7 to 45.0)	29.1 (18.9)	0.928^d^
Total length of delivery (Minute)	270.0 (190.0 to 355.0)	274.5 (141.5)	300.0 (210.0 to 390.0)	296.9 (135.0)	0.520^c^
Pain	6.5 (5.0 to 7.4)	6.2 (1.4)	7.8 (7.0 to 8.5)	7.5 (1.4)	< 0.001^d^

^a^Median (Percentile 25 to Percentile 75)

^b^Standard Deviation

^c^Independent t-test

^d^Mann Whitney U

There was no significant difference between women with gravida 1 and 2 in terms of mean severity of labor pain (*P* = 0.818) and post-intervention mean score of perceived stress (*P* = 0.338).

### Secondary outcomes

The preintervention mean (SD) score of childbirth fear was 38.3 (22.9) in the distraction techniques group and 46.3 (17.1) in the control group. Also, in the 36th week of gestation, it was 29.1 (18.9) in the distraction techniques group and 39.1 (29.6) in the control group. There was no statistically significant difference between the two groups before the intervention (*P* = 0.117), but in the 36th week of pregnancy, according to ANCOVA test with adjusting the preintervention score, the mean score of the fear of childbirth in the distraction techniques group was less than that of the control group, but the difference was not statistically significant (AMD: 5.4; 95% CI: − 2.4 to 13.0; *P* = 0.117) (Table 2).

The duration of the active phase (*P* = 0.504), the second stage of delivery (*P* = 0.928), and the total duration of delivery (*P* = 0.520) were lower in the intervention group, but this difference was not statistically significant (Table 3). There was no significant difference between the two groups in terms of the first minute Apgar score (*P* = 1.000) and the frequency of oxytocin consumption (*P* = 0.622) (Table 4).

**Table 4:** Comparison of Apgar score and receiving oxytocin between two groups of the distraction technique and control

	Distraction techniques (*N* = 34) Number (percent)	Control (*N* = 34) Number (percent)	*P*-value
Apgar at the first minute			1.000^b^
7	1 (29)	0 (0)	
8	2 (5.9)	3 (9.4)	
9	29 (85.3)	28 (87.5)	
10	2 (5.9)	1 (3.1)	
Receiving of oxytocin			0.622^a^
Yes	15 (45.5)	16 (51.6)	
No	18 (54.5)	15 (48.4)	

^a^Chi-square test

^b^Fisher’s exact test

## Discussion

This is the first study to examine the effect of distraction techniques on stress and labor pain as primary outcomes and fear of childbirth and the length of delivery stages as secondary outcomes. The results of this study showed that the mean stress score and labor pain in the distraction techniques group was significantly less than that of the control group. Also, the mean of fear of childbirth at 36 weeks of gestation, duration of the active phase and second stage of delivery, and total length of delivery in the distraction techniques group were less than the control group; however, this difference was not statistically significant. First minute Apgar score and oxytocin consumption were not significantly different between the two groups.

The results of this study showed the effect of distraction techniques on reducing labor pain. As discussed earlier, given the lack of a study in this area, therefore the results of other studies that investigated the effects of distraction techniques on pain control during bronchoscopy [37], physiotherapy in burn patients [38], LP pain in cancer patients [39] were reported to confirm the findings of this study. Diette et al. studied the effect of distraction techniques on pain intensity during bronchoscopy on 80 patients in two groups. The results showed that the intervention group had a significant reduction in the pain score compared to the control group [37]. Hoffman et al., in a clinical trial, studied the effect of distraction techniques on 12 patients aged 19 to 47 who had burned on average 21%. The results showed that pain score was significantly was less when using the distraction technique [38]. In another study, the effect of the distraction technique has been assessed on pain severity during LP (lumbar puncture) in cancer patients. The results of this study showed that the pain score of the intervention group was significantly less than that of the control group [39]. The results of the above studies on pain control using distraction techniques are consistent with the results of this study.

In this study, the mean score of perceived stress in the intervention group was less than that control group. In a randomized controlled clinical trial that examined the effect of distraction on salivary cortisol levels after acute stress, Salzmann et al. showed lower levels of cortisol and alpha-amylase (stress markers) in the distraction group than the other two groups [40]. Also, the results of a review study that evaluated the use of distraction techniques in obstetrics and gynecology showed that this technique was effective in reducing stress [30]. The results of this study were in line with the present study.

The results of this study showed the effect of distraction techniques on reducing fear of childbirth. In a clinical trial aimed at the effect of distraction techniques on fear, 50 children and teenager aged 5 to 18 years old were divided into two groups of intervention (22 patients) and control (28 patients). The fear score of the intervention group was significantly reduced compared to the control group [28]. In another study, the effect of video stimulus films on fear in adult’s patients receiving hyperbaric oxygen therapy was investigated. The results showed that the fear score in the intervention group was significantly less than the control group [29]. The results of these two studies on reducing fear are not consistent with the results of the present study. This inconsistency may be due to differences in the time of measurement. The fear score in the present study was measured prior to the onset of labor pain and at 36 weeks gestation, while it was measured in the mentioned studies during therapeutic interventions. In addition, the fear factor and duration of exposure to the agent, as well as the patterns of the person’s fear and even the degree of fear measurement in these studies varied.

In the present study, there was no statistically significant difference between the two groups in terms of the active phase of labor, second stage of delivery and total length of delivery, Apgar score and oxytocin consumption. Based on search conducted by researcher, no study was found regarding the effect of distraction techniques on the mentioned outcomes. The mean duration of the normal active phase of labor for nulliparous women is 4.9 h and the median duration of second phase of delivery is about 50 min for nulliparous women and approximately 20 min for multiparous women [2]. In the present study, the duration of active phase was 4.1 h in the intervention group and 4.5 h in the control group as well as the median duration of second phase of delivery was 25 min in the intervention group and 30 min in the control group which are approximately consistent with the normal length of the active and second phases of delivery. In a trial, the effect of continuous labor support by midwife has been assessed on delivery outcomes and it has been shown that the oxytocin consumption, Apgar score and duration of active phase were not significantly different between the two intervention and control groups [41]. The results of this study are in line with the present study.

### Strengths and limitations

One of the strengths of this study is the implementation of distraction techniques intervention on labor pain and stress, for the first time, and all the principles of clinical trial, including random allocation, and allocation concealment, were observed to prevent selection bias. Existence of a private room for counseling in health care centers and a single LDR (Labor, Delivery and Recovery) room in the hospital delivery unit and also staff and obstetricians collaboration were another strengths of this study. Using standard questionnaires to measure perceived stress, pain, and fear of childbirth are other strengths of this study.

One of the limitations of this research is that it was conducted only on women with first and second pregnancies, therefore, the results cannot be generalized to women with third or higher pregnancies. Moreover, considering that the intervention and data collection was done by the first author, there was no possibility of blinding of participants and data collector. It is suggested that the effect of distraction techniques on the severity of fear and stress in patients before cesarean section, Posttraumatic Stress Disorder (PTSD) and etc. to be studied.

## Conclusion

Based on the results of this study, the distraction techniques can be useful as an easy, inexpensive, and available method to reduce the stress and pain during labor. However, more clinical trials are needed to confirm the effectiveness of distraction techniques.

## Data Availability

Datasets used and analyzed during this study are available from the corresponding author on reasonable request.

## Abbreviations

95% CI: 95% Confidence Interval
AMD: Adjusted Mean Difference
ANCOVA: Analysis of covariance
IUD: Intra Uterine Device
PSS: Perceived Stress Scale
SD: Standard Deviation
VAS: Visual Analogue Scale
W-DEQ: Wijma Delivery Expectancy/Experience Questionnaire
